# Coexisting patterns and significance of serum HBV RNA and HBV DNA in patients with treatment-naïve chronic hepatitis B virus infection

**DOI:** 10.3389/fmed.2024.1525476

**Published:** 2025-01-08

**Authors:** Kun-yan Hao, Ye Fan, Yi-qing Zhang, Yue-cheng Yu

**Affiliations:** ^1^Center of Hepatology and Department of Infectious Disease, Jinling Hospital Affiliated to School of Medicine, Nanjing University, Nanjing, China; ^2^State Key Laboratory of Organ Failure Research, Guangdong Province key Laboratory of Viral Hepatitis Research, Department of Infectious Diseases, Nanfang Hospital, Southern Medical University, Guangzhou, China; ^3^Department of Clinical Laboratory, Jinling Hospital Affiliated to School of Medicine, Nanjing University, Nanjing, China

**Keywords:** HBV RNA, HBV DNA, HBsAg, HBeAg, coexisting patterns, treatment-naïve HBV-infectors

## Abstract

**Aim:**

The study aimed to explore the coexisting patterns and assess the significance of serum hepatitis B virus (HBV) RNA and traditional virological biomarkers in patients with antiviral treatment-naïve chronic hepatitis B virus (HBV) infection.

**Methods:**

Serum HBV RNA, HBV DNA, hepatitis B surface antigen (HBsAg), and hepatitis B envelope antigen (HBeAg) levels were measured and compared in patients with chronic hepatitis B virus infection. The HBV RNA levels were determined using a simultaneous amplification and testing assay.

**Results:**

In the HBeAg-negative (HBeAg [−]) patients, the serum HBV RNA detectable (HBV RNA [+]) rate (33.33%, 14/42) was significantly lower than the serum HBV DNA detectable (HBV DNA [+]) rate (95.24%, 40/42; *p* < 0.001). However, there was no significant difference in the HBeAg-positive (HBeAg [+]) patients (*p* > 0.05). The HBV RNA (+) rate (33.33%, 14/42) was lower in the HBeAg-negative patients than in the HBeAg-positive patients (100%, 17/17, *p* < 0.001), while the HBV DNA (+) rate (95.24%, 40/42 vs. 94.12%, 16/17) showed no significant difference (*p* > 0.05). The HBV RNA (+) rates showed a significant difference (*p* < 0.001) among the different HBsAg levels (10.00, 65.00, 84.21%, *p* < 0.001), while the HBV DNA (+) rate showed no significant difference (*p* > 0.05). In all patients, serum HBV RNA correlated well with HBV DNA (*r* = 0.72, *p* < 0.001), HBeAg (*r* = 0.68, *p* < 0.001), and HBsAg (*r* = 0.66, *p* < 0.001). However, the correlations between HBV RNA and other biomarkers varied across the different HBsAg and HBeAg levels.

**Conclusion:**

The coexisting patterns of serum HBV RNA and HBV DNA varied with the levels of HBeAg and HBsAg in the patients with treatment-naïve chronic HBV infection. This further suggests that serum HBV RNA should be included in the key index panel to accurately evaluate the natural history of HBV infection and the effects of antiviral treatment.

## Introduction

The global prevalence of hepatitis B surface antigen (HBsAg) in all age groups was 4.1% in 2019 (1), which remains a significant worldwide health problem, leading to cirrhosis, liver cancer, and liver-related mortality. Therefore, accurate and timely management and treatment of hepatitis B virus (HBV) have important medical and social significance. In the past, we primarily relied on traditional serological biomarkers to assess HBV infection, including HBV DNA, hepatitis B surface antigen (HBsAg), hepatitis B envelope antigen (HBeAg), and alanine aminotransferase (ALT). According to the abovementioned traditional biomarkers, HBeAg-negative (HBeAg [−]) chronic HBV-infected patients with normal ALT and low HBV DNA levels (< 2000 IU/mL) or HBeAg-positive (HBeAg [+]) patients with high HBV DNA load and normal ALT levels are mostly considered inactive carriers or immune-tolerant individuals who do not require treatment. However, many of these patients were also found to have inflammation or fibrosis, confirmed by histology (2, 3). Hence, in both HBeAg-positive and HBeAg-negative patients, traditional serological biomarkers are not sufficient to provide guidance on prognosis, monitor virus activity, or determine antiviral treatment accurately and in a timely manner in patients with treatment-naïve HBV. Covalently closed circular DNA (cccDNA) is considered the most direct biomarker for assessing the profile of HBV infection. However, the need for a liver biopsy, along with the lack of a standardized method to quantify cccDNA, prevents its widespread use in clinics. HBV RNA is one of the transcripts from cccDNA and has been considered a new biomarker of cccDNA (4, 5), which can be detected in serum. Unfortunately, it is still unclear whether these viral serum markers can replace each other, indicating the need for further studies on the applications of serum HBV RNA. Therefore, in this study, we focused on the serum HBV RNA detectable (HBV RNA [+]) rate and level and explored the coexisting patterns of serum HBV RNA, HBV DNA, HBsAg, and HBeAg in patients with treatment-naïve HBV who had normal ALT and total bilirubin (TBil).

## Methods

### Patients

Based on the clinical timeline, we prospectively recruited 59 treatment-naïve chronic HBV-infected individuals between October 2020 and June 2022 at the Department of Hepatology and the Department of Infectious Diseases, Jinling Hospital, affiliated with the School of Medicine, Nanjing University, Nanjing, China The study complied with Good Clinical Practice and the Declaration of Helsinki and was approved by the Ethics Committee of the hospital (number: DZQH-KYLLFS-20-01). All participants provided written informed consent for the future analysis of the samples collected during the study. The inclusion criteria were as follows: serological HBsAg positivity for at least 6 months, age over 18 years, normal ALT and TBil levels, and antiviral treatment-naïve status. The exclusion criteria were as follows: pregnancy, history of carcinoma, and coinfection with other viruses or diseases.

### Serum HBV RNA concentrations

The HBV RNA levels were determined by employing an RNA simultaneous amplification testing method (HBV-SAT) based on real-time fluorescence detection of 42°C isothermal RNA amplification using the HBV-SAT kit (Shanghai Rendu Biotechnology Co., Ltd. China) (6). The kit was registered with China’s National Medical Products Administration (NMPA) on 15th March 2021 and officially approved for clinical use. The target RNA levels were reverse transcribed using the Moloney murine leukemia virus enzyme, transcribed using T7 RNA polymerase, and detected using an RNA molecular beacon probe labeled with fluorescence and a quencher. The RNA extraction, amplification, and detection were performed using an automated AutoSAT system (Shanghai Rendu Biotechnology Co., Ltd. China). An internal calibrator/internal control (IC) was added to each reaction. The concentration of a sample was determined using the HBV and IC signals for each reaction, and these were compared with calibration information. The linear range was from 1 × 10^2^ to 1 × 10^8^ copies/ml. The lowest limit of detection (LLD) was 50 copies/ml, but the values between 50 and 100 copies/ml were not linear. The R2 value of the linear equation was greater than 0.95. If the value was <50 copies/ml, the serum HBV RNA level was considered undetectable (HBV RNA [−]), and if the value was ≥50copies/ml, the serum HBV RNA level was considered detectable (HBV RNA [+]). As the linear quantitative LLD of the serum HBV RNA in our method was 100 copies/ml, the samples with HBV RNA < 100 copies/ml were assigned a value of 50 copies/ml for correlation analysis, as reported by Zhang Wen-hong et al. (7).

### Serum HBV DNA concentrations

The serum HBV DNA levels were determined using a fluorogenic quantitative PCR assay (TIANLONG Biotech Co. Ltd., Suzhou, China). The linear range was from 30 to 1 × 10^8^ IU/mL. The LLD value was 10 IU/mL, but the values between 10 and 30 IU/mL were not linear. If the value was <10 IU/mL, the serum HBV DNA level was considered undetectable (HBV DNA [−]), and if the value was ≥10 IU/mL, the serum HBV DNA level was considered detectable (HBV DNA [+]). As the linear quantitative LLD of the serum HBV DNA in our method was 30 IU/mL, the samples with HBV DNA < 30 IU/mL were assigned a value of 15 IU/mL for correlation analysis, as reported by Zhang Wen-hong et al. (7).

### Serum HBsAg and HBeAg quantification

The HBsAg and HBeAg levels were quantified using the ARCHITECT assay (Abbott Laboratories, Illinois, USA), with the HBsAg dynamic range from 0.05 to 125,000 IU/mL.

### Serum liver function test

The liver function was tested using the Siemens ADVIA 2400 automatic biochemical instrument (Siemens, Germany), with the reagent provided by the matching kit from Siemens.

### Statistical analyses

The chi-square test or Fisher’s exact test was used to determine differences between categorical variables. Continuous normally distributed variables were tested using Student’s *t-*test. Correlations were assessed using the non-parametric Spearman test. The analyses were carried out using SPSS software version 23.0. All tests for significance and the resulting *p-*values were two-sided and considered significant if the *p-*value was <0.05.

## Results

### Patient demographics and baseline characteristics

In this study, 59 patients with treatment-naïve HBV were enrolled. Their ages ranged from 21 to 70 years (40.93 ± 10.21, mean value = 40.93). The male-to-female ratio was 2.47 (42/17). Of the participants, 17 were HBeAg positive and 42 were HBeAg negative. Only three patients were serum HBV DNA negative, and one of these three had a low level of HBV RNA (62.1copies/ml, HBV RNA [+]). The remaining 56 patients were all serum HBV DNA positive, with 30 of the 56 patients (53.57%) being serum HBV RNA positive, while 26 patients had undetectable HBV RNA (Table 1).

**Table 1:** Overall characteristics of the patients with untreated HBV.

Characteristic	Total
Case numbers (*n*)	59
Age (year)	40.93 ± 10.21
Sex, male/female (male%)	42/17 (71.19)
HBV DNA (IU/mL), median (IQR)	1,648 (451.20,3,934,000)
HBV DNA (+) rate (%)	56/59 (94.92)
HBV RNA (IU/mL), median (IQR)	50 (50,3,740,000)
HBV RNA (+) rate (%)	31/59 (52.54)

### Coexisting patterns of serum HBV RNA and serum HBV DNA

In all 59 patients with treatment-naïve HBV, the serum HBV RNA (+) rate (52.54%, 31/59) was significantly lower than the serum HBV DNA rate (94.92%,56/59; *χ*^2^ = 25.20, *p* < 0.001).

In 31 HBV RNA-positive patients, the HBV DNA (+) and HBV DNA (−) rates were 96.77% (30/31) and 3.23% (1/31), respectively. In 28 HBV RNA-negative patients, the HBV DNA (+) and HBV DNA (−) rates were 92.86% (26/28) and 7.14% (2/28), respectively.

**Figure 1:**
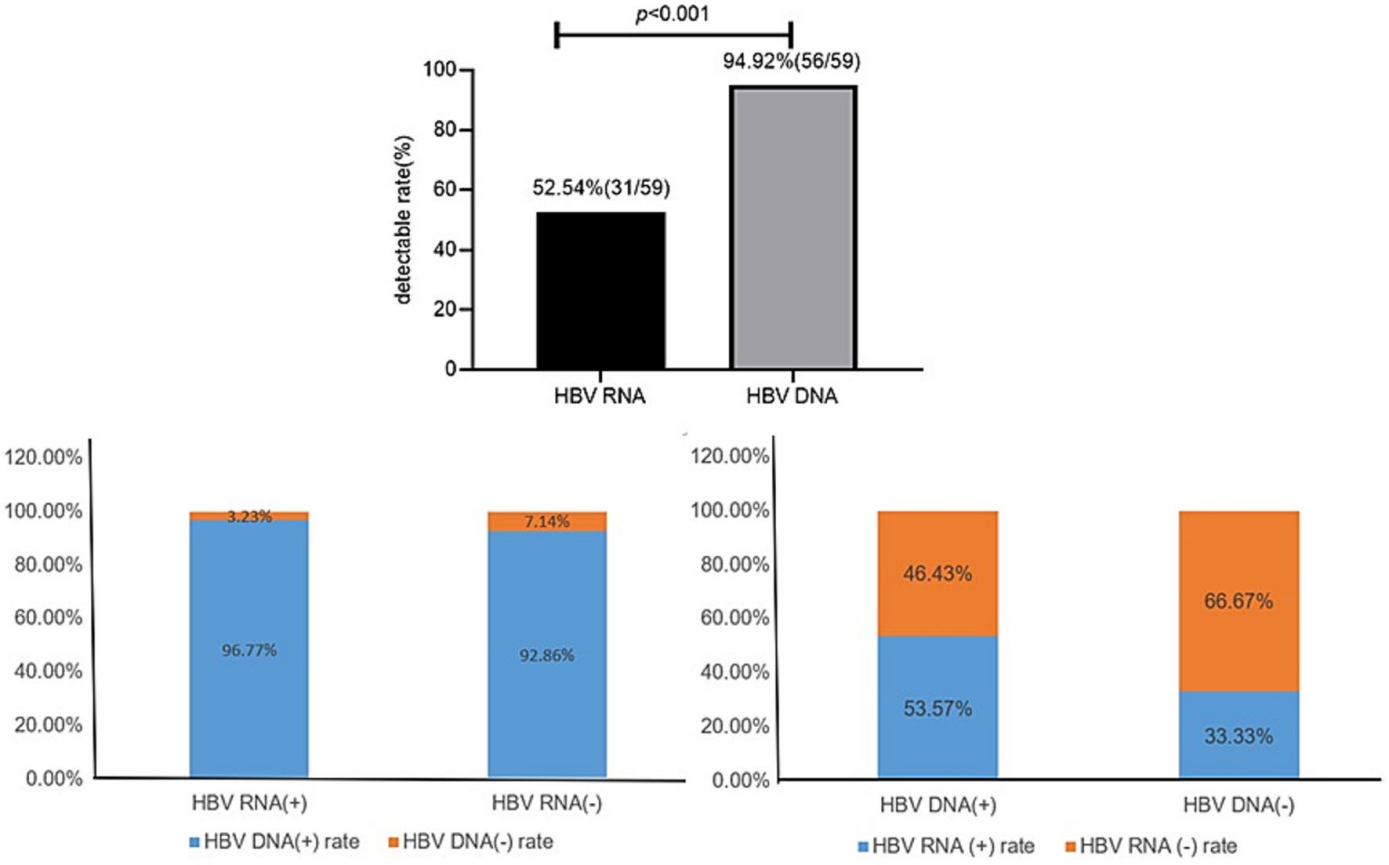
Coexisting patterns of HBV RNA and HBV DNA.

In the 56 HBV DNA-positive patients, the HBV RNA (+) and HBV RNA (−) rates were 53.57% (30/56) and 46.43% (26/56), respectively. In the three HBV DNA-negative patients, the serum HBV RNA (+) and HBV RNA (−) rates were 33.33% (1/3, 62.10copies/ml) and 66.67% (2/3), respectively (Figure 1).

### HBV RNA and HBV DNA detectable rate in the different HBeAg statuses

In the HBeAg-negative patients, the serum HBV RNA (+) rate (33.33%, 14/42) was significantly lower than the serum HBV DNA (+) rate (95.24%, 40/42; *χ*^2^ = 32.41, *p* < 0.001). However, in the HBeAg-positive patients, the serum HBV RNA (+) (100%, 17/17) and serum HBV DNA (+) (94.12%, 16/17) rates showed no significant difference (*p* > 0.05).

**Figure 2:**
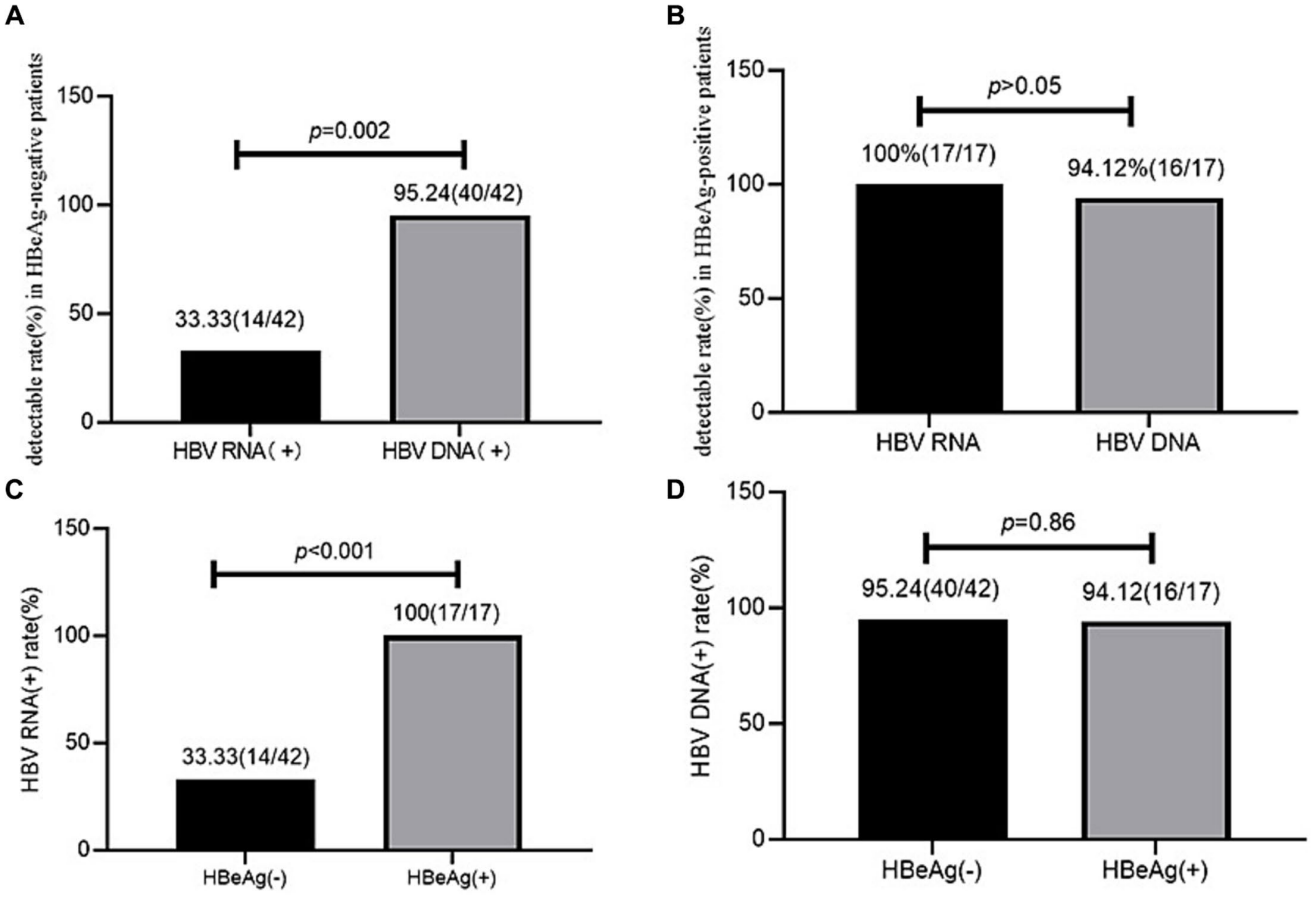
HBV RNA and HBV DNA detectable rate in HBeAg positive and negative patients. **(A)** HBV RNA and HBV DNA detectable rates in the HBeAg-negative patients. **(B)** HBV RNA and HBV DNA detectable rates in the HBeAg-positive patients. **(C)** Comparison of the HBV RNA detectable rates between the HBeAg-positive and HBeAg-negative patients. **(D)** Comparison of the HBV DNA detectable rates between the HBeAg-positive and HBeAg-negative patients.

The HBV RNA (+) rate (33.33%, 14/42) was lower in the HBeAg-negative patients than in the HBeAg-positive patients (100%, 17/17, χ^2^ = 18.98, *p* < 0.001), while the HBV DNA (+) rate (95.24%, 40/42 vs. 94.12%, 16/17) showed no significant difference between the HBeAg-negative and HBeAg-positive patients (*χ*^2^ = 0.03, *p* = 0.86) (Figure 2).

### Relationship between HBV RNA and HBV DNA in the different HBsAg statuses

We compared the HBV RNA and HBV DNA (+) rates and levels across the different HBsAg statuses. We divided all 59 patients into three groups: 20 patients with HBsAg<100 IU/mL, 20 patients with 100 ≤ HBsAg < 5,000 IU/mL, and 19 patients with HBsAg>5,000 IU/mL. The HBV RNA (+) rates were 10.00, 65.00, and 84.21%, respectively, with a significant difference (*p* < 0.001) among the three groups, while the HBV DNA (+) rate showed no significant difference (*p* > 0.05) (Table 2).

**Table 2:** Serum HBVRNA and HBV DNA detectable rates and levels across the different HBsAg statuses (IU/ml).

Items	HBsAg<100 (*n* = 20)	100 ≤ HBsAg ≤ 5,000 (*n* = 20)	HBsAg>5,000 (*n* = 19)	*χ^2^*	*p*-value
HBVRNA (+) rate (%)	10.00 (2/20)	65.00 (13/20)	84.21 (16/19)	26.16	<0.001^*^
HBVDNA (+) rate (%)	90.00 (18/20)	95.00 (19/20)	100.00 (19/19)	/	0.16

*p denotes significant differences among the three groups.

### Mutual correlations between serum HBV RNA and the other biomarkers

In all 59 patients with HBV, serum HBV RNA correlated well with HBV DNA (*r* = 0.72, *p* < 0.001), HBeAg (*r* = 0.68, *p* < 0.001), and HBsAg (*r* = 0.66, *p* < 0.001). However, the correlations differed in the subgroup analysis according to the different HBsAg and HBeAg levels (Table 3).

**Table 3:** Correlation coefficients between serum HBV RNA and the other viral biomarkers in all 59 patients.

Variables	HBV DNA	HBsAg	HBeAg
HBV RNA	*r* = 0.72	*r* = 0.66	*r* = 0.68
	(*p*<0.001)	(*p*<0.001)	(*p*<0.001)

In the 17 HBeAg-positive patients, serum HBV RNA correlated only with HBeAg (*r* = 0.54, *p* = 0.02) and not with HBV DNA (*r* = 0.16, *p* = 0.53) or HBsAg (*r* = 0.45, *p* = 0.07). When HBeAg >1,500 S/Co, HBV RNA correlated well with HBsAg (*r* = 0.87, *p* = 0.02) and HBeAg (*r* = 0.98, *p* < 0.001). When HBsAg > 10,000 IU/mL, HBV RNA only correlated well with HBsAg (*r* = 0.93, *p* < 0.001) and HBeAg (*r* = 0.64, *p* = 0.04). However, these correlations were not observed at other HBsAg and HBeAg levels (Table 4).

**Table 4:** Correlation coefficients between serum HBV RNA and the other viral biomarkers in the HBeAg-positive patients.

Variables		HBV DNA	HBsAg	HBeAg
HBV RNA	All	*r* = 0.16	*r* = 0.45	*r* = 0.54
	(*n* = 17)	(*p* = 0.53)	(*p* = 0.07)	(*p* = 0.02)
	HBeAg (S/Co)			
	HBeAg<1,500 (*n* = 11)	*r* = 0.41	*r* = 0.06	*r* = 0.48
	(932.84 ± 608.79)	(*p* = 0.22)	(*p* = 0.85)	(*p* = 0.13)
	HBeAg>1,500 (*n* = 6)	*r* = −0.53	*r* = 0.87	*r* = 0.98
	(1647.28 ± 146.45)	(*p* = 0.28)	(*p* = 0.02)	(*p* < 0.001)
	HBsAg (IU/ml)			
	HBsAg<10,000 (*n* = 7)	*r* = 0.45	*r* = 0.21	*r* = 0.61
	(3117.43 ± 2107.64)	(*p* = 0.32)	(*p* = 0.65)	(*p* = 0.15)
	HBsAg>10,000 (*n* = 10)	*r* = 0.02	*r* = 0.93	*r* = 0.64
	(50153.21 ± 31199.09)	(*p* = 0.96)	(*p* < 0.001)	(*p* = 0.04)

In the 42 HBeAg-negative patients, HBV RNA correlated with HBsAg (*r* = 0.39, *p* = 0.01) but not with HBV DNA (*r* = 0.17, *p* = 0.27) or HBeAg (*r* = 0.09, *p* = 0.58). When the cutoff value for the serum HBsAg level was 1,000 IU/mL, serum HBV RNA did not correlate with HBV DNA, HBsAg, and HBeAg, neither when HBsAg < 1,000 IU/mL nor when HBsAg > 1,000 IU/mL (*p* > 0.05). The same was true when the cutoff value was 1,500 IU/mL. However, serum HBV RNA correlated with HBeAg (r = 0.66, p = 0.02) only when serum HBsAg was more than 3,000 IU/mL (Table 5).

**Table 5:** Correlation coefficients between HBV RNA and the other viral biomarkers in the HBeAg(−) patients.

Variables		HBV DNA	HBsAg	HBeAg
HBV RNA	All (*n* = 42)	*r* = 0.17	*r* = 0.39	*r* = 0.09
		(*p* = 0.27)	(*p* = 0.01)	(*p* = 0.58)
	HBsAg (IU/ml)			
	HBsAg<1,000 (*n* = 23)	*r* = 0.07	*r* = −0.08	*r* = 0.05
	(57.09 ± 96.07)	(*p* = 0.74)	(*p* = 0.71)	(*p* = 0.81)
	HBsAg>1,000 (*n* = 19)	*r* = 0.07	*r* = 0.30	*r* = 0.37
	(5725.09 ± 4257.04)	(*p* = 0.78)	(*p* = 0.22)	(*p* = 0.12)
	HBsAg<1,500 (*n* = 25)	*r* = 0.02	*r* = −0.11	*r* = 0.07
	(154.06 ± 352.90)	(*p* = 0.91)	(*p* = 0.59)	(*p* = 0.75)
	HBsAg>1,500 (*n* = 17)	*r* = 0.17	*r* = 0.18	*r* = 0.42
	(6249.31 ± 4196.28)	(*p* = 0.25)	(*p* = 0.49)	(*p* = 0.09)
	HBsAg<3,000 (*n* = 30)	*r* = −0.07	*r* = 0.25	*r* = 0.00
	(498.37 ± 860.32)	(*p* = 0.40)	(*p* = 0.94)	(*p* = 1.00)
	HBsAg>3,000 (*n* = 12)	*r* = 0.27	*r* = −0.23	*r* = 0.66
	(7928.22 ± 3885.20)	(*p* = 0.40)	(*p* = 0.47)	(*p* = 0.02)

## Discussion

Although serum HBV RNA has been recognized as a new biomarker of cccDNA (4, 5), the clinical usefulness of serum HBV RNA is not fully understood, especially in different phases of HBV infection. In our study of 59 treatment-naïve chronic HBV-infected individuals with normal ALT and TBil levels, the rate of detectable serum HBV RNA was lower than that of serum HBV DNA in the HBeAg-negative patients. However, the rates of HBV RNA (+) and HBV DNA (+) showed no significant difference in the HBeAg-positive patients. The HBV RNA (+) rate was lower in the HBeAg-negative patients than in the HBeAg-positive patients, while the HBV DNA (+) rate showed no significant difference between the two groups. The HBV RNA (+) rate showed significant differences among the patients with different levels of HBsAg, while the HBV DNA (+) rate did not show any significant differences. In addition, the correlations between HBV RNA and the other biomarkers varied depending on the HBeAg status and HBsAg levels in the treatment-naïve HBV-infected patients.

In our study, we found that only three patients were serum HBV DNA negative, and one of the three patients had a low level of HBV RNA (62.1copies/ml, HBV RNA [+]). The remaining 56 patients were all serum HBV DNA positive, with 30 of the 56 patients (53.57%) being serum HBV RNA positive, while 26 patients had undetectable HBV RNA. Therefore, even without calculating the specificity and sensitivity of serum HBV RNA, the results are sufficient to show that it is clinically more meaningful to detect HBV RNA and HBV DNA simultaneously than to detect HBV DNA alone.

In our study, one of the three HBV DNA-negative patients had a low and non-linear level of HBV RNA (62.1copies/ml). It is likely that the viral replication in this patient was very weak due to a limited number of HBV RNA templates and/or insufficient RNA-dependent DNA polymerase (RDDP) reverse transcriptase (RT) activity, which was not enough to transfer more HBV DNA for detection in the serum. On the other hand, why did 26 HBV DNA (+) patients have undetectable serum HBV RNA? The reasons may lie in two aspects. Firstly, there were traces of hard-to-detect HBV RNA in the liver, but the HBV RT activity was high enough to reverse transcribe sufficient detectable serum HBV DNA. Secondly, serum HBV DNA may come from incomplete HBV RNA fragments transcribed from integrated HBV S gene fragments, and such fragmented HBV RNA is difficult to detect with the current kits, which are designed to detect the full length of HBV RNA (8). Therefore, these aspects warrant further investigation in the future.

Our study showed that in all 59 patients with treatment-naïve HBV, the serum HBV RNA (+) rate (52.54%) was significantly lower than the serum HBV DNA rate (94.92%). This trend was also observed in the HBeAg-negative patients (33.33% vs. 95.24%, *p* < 0.001). However, in the HBeAg-positive patients, the serum HBV RNA (+) and HBV DNA (+) rates showed no significant difference (100% vs. 94.12%, *p* > 0.05). These findings are in line with those of previous studies (9, 10). Furthermore, we found that the serum HBV RNA (+) rate was higher in the HBeAg-positive patients (100%) than in the HBeAg-negative patients (33.33%, *p* < 0.001), which aligns with the reports of previous studies (7, 11, 12). However, the serum HBV DNA (+) rate showed no significant difference between the HBeAg-positive and HBeAg-negative patients. It has been reported that HBV transcriptional activity, measured as the amount of HBV RNA per cccDNA molecule, is higher in HBeAg-positive patients compared to HBeAg-negative patients, leading to more HBV RNA in HBeAg-positive patients. HBV RNA can only be transcribed from cccDNA, while serum HBV DNA can be transcribed from both cccDNA and integrated chromosomes. The amount of integrated chromosomes was higher in the HBeAg-negative participants than in the HBeAg-positive participants. As a result, the integrated chromosome contributed to serum HBV DNA, especially in the HBeAg-negative patients, which accounted for the lack of difference in serum HBV DNA between the HBeAg-positive and HBeAg-negative patients (8).

The HBV RNA (+) rate showed a significant difference across the different HBsAg levels, with higher HBsAg levels correlating to a higher HBV RNA (+) rate, while the HBV DNA (+) rate showed no statistical difference. Both HBsAg and HBV DNA may not only originate from cccDNA but also from integrated HBV sequences (8, 13, 14). Rather than being produced by a viral integrant, serum HBV RNA is only produced by cccDNA, reflecting the direct viral transcriptional activity of cccDNA (4, 5).

In general, the HBV RNA levels varied according to the HBeAg and HBsAg statuses. Meanwhile, serum HBV RNA and serum HBV DNA dissociated in reflecting HBV activity in the patients with treatment-naïve HBV.

The correlations between serum HBV RNA and traditional biomarkers found in our study are partially similar to those in other studies. In our study, serum HBV RNA correlated well with serum HBV DNA, HBsAg, and HBeAg in all 59 patients with treatment-naïve HBV, which is consistent with findings from another report (6). However, the correlations differed across the different HBsAg and HBeAg statuses. We found that in the HBeAg-positive patients, HBV RNA only correlated with HBeAg. Further analysis revealed that serum HBV RNA correlated with HBeAg and HBsAg only when HBeAg>1500S/CO or HBsAg>10,000 IU/mL. Although in the HBeAg-negative patients, HBV RNA correlated with HBsAg, the correlation value was low (*r* = 0.39), consistent with other reports (9, 11). In addition, another study showed no correlation between HBV RNA and HBsAg in HBeAg-negative patients (7). Based on the different HBsAg levels, we found that only when serum HBsAg>3,000 IU/mL, HBV RNA correlated with HBeAg. These findings indicate that the correlations between HBV RNA and the other biomarkers varied according to the replication activity of HBV, with stronger correlations associated with higher replication activity. In contrast, other reports showed that serum HBV RNA correlated with serum HBV DNA both in HBeAg-positive and HBeAg-negative patients (6, 9, 11). The differences may be attributed to factors such as integrations, genotypes, and the laboratory methods used to measure HBV RNA. Firstly, it was reported that 87% of the total intrahepatic HBV DNA in HBeAg-negative patients was derived from its integration into the host hepatocytes chromosome, compared to 46% in HBeAg-positive patients (*p* < 0.001) (12). Full-length HBV RNA cannot be transcribed from integrated HBV DNA fragments in hepatocyte chromosomes, but integrated HBV DNA can guide the synthesis of HBsAg. In HBeAg-negative patients, HBsAg is believed to be mainly synthesized from the HBV genome integrated into the host chromosomes, rather than from cccDNA (12). Secondly, the relative transcriptional activity of the integrated HBV-containing opening reading frame (ORF) S was higher in the patients with HBeAg-negative chronic hepatitis compared to those with HBeAg-positive chronic hepatitis. The transcriptional activity of ORF S from integrated HBV was higher than that of ORF X in HBeAg-negative chronic hepatitis (13), which could explain why the serum HBsAg levels were more influenced by the HBeAg status. Thirdly, differences in serum HBV RNA quantification methods between our study and others also contributed to the variation in results (6). Fourthly, although other studies showed correlations between HBV RNA and HBsAg in HBeAg-negative patients, the correlation was weaker in HBeAg-negative patients compared to HBeAg-positive patients (9, 11). The correlation was so weak that it disappeared in certain situations, as observed in our study (7). Serum HBV RNA showed a distinct profile among patients with HBV across different immune statuses and hepatic histopathology stages (12). The clinical utility of HBV RNA may vary in different phases of the disease (6), serving as a supplement to traditional indicators.

## Conclusion

In the patients with treatment-naïve HBV, the serum HBV RNA (+) rate differed from the serum HBV DNA rate. The HBV RNA (+) rate was higher in the HBeAg-positive patients compared to the HBeAg-negative patients and varied across the different HBsAg levels. The correlations between HBV RNA and the traditional biomarkers varied depending on the different levels of HBV replication activity (higher replication activity led to stronger correlations). This variation may primarily be due to the integrated genome, which influences the levels of traditional serological biomarkers. To summarize, the coexisting patterns of serum HBV RNA and HBV DNA varied with the levels of HBeAg and HBsAg in the patients with treatment-naïve chronic HBV infection. This suggests that serum HBV RNA should be included in the key diagnostic panel to more accurately assess the natural history of HBV infection and the effects of antiviral treatment.

### Limitations

In our study, we only included a small number of HBeAg-positive individuals (7) and our patients did not encompass all types of HBV infections. Larger-scale studies are needed to validate our findings. In addition, we did not assess the integrated genome, genotypes, mutations, cccDNA, or intrahepatic HBV DNA, which could have provided a deeper understanding of the correlations.

## Data Availability

The original contributions presented in the study are included in the article/supplementary material, further inquiries can be directed to the corresponding author.
